# Endovascular treatment of diffuse mandibular arteriovenous malformation: a case report and literature review

**DOI:** 10.3389/fcvm.2025.1640062

**Published:** 2026-01-08

**Authors:** Defu Dong, Kai Zheng, Bo Ma, Qiangqiang Nie, Xueqiang Fan, Peng Liu, Zhidong Ye

**Affiliations:** 1 Shanxi Bethune Hospital, Shanxi Academy of Medical Sciences, Third Hospital of Shanxi Medical University, Tongji Shanxi Hospital, Taiyuan, China; 2 China-Japan Friendship Hospital (Institute of Clinical Medical Sciences), Chinese Academy of Medical Sciences & Peking Union Medical College, Beijing, China; 3 Department of Cardiovascular Surgery, China-Japan Friendship Hospital, Beijing, China

**Keywords:** coil embolization, endovascular treatment, intra-osseous arteriovenous malformation, mandibular arteriovenous malformation, sclerotherapy

## Abstract

**Background:**

Arteriovenous malformations (AVMs) are rare congenital vascular anomalies involving high-flow shunting between arteries and veins. Intraosseous AVMs of the mandible are particularly uncommon and present substantial diagnostic and therapeutic challenges. Because even minor trauma or dental procedures may trigger severe hemorrhage, early recognition and appropriate management are essential.

**Case presentation:**

We describe a 33-year-old woman with a long-standing vascular lesion of the right mandible who developed uncontrolled oral bleeding after tooth extraction. Imaging confirmed a diffuse intraosseous AVM. After multiple unsuccessful treatments elsewhere, she underwent endovascular therapy consisting of super-selective arterial and venous access, coil embolization, and sclerotherapy with absolute ethanol and polidocanol foam. The procedure resulted in complete occlusion of the lesion without complications. Follow-up at 4 days, 6 months, and 1 year showed no recurrence.

**Conclusion:**

Mandibular AVMs are complex lesions that carry a significant risk of life-threatening hemorrhage. This case demonstrates that combined endovascular embolization and sclerotherapy can provide effective and durable control. Accurate diagnosis, timely intervention, and continued follow-up are essential to achieving favorable outcomes for this rare condition.

## 1 Introduction

Arteriovenous malformations (AVMs) are uncommon congenital vascular abnormalities characterized by direct high-flow connections between arteries and veins (1). They consist of enlarged vessels without a normal capillary bed. AVMs that occur inside the jawbone, known as intraosseous arteriovenous malformations of the jaw (JAVMs), are even rarer in clinical practice (2). Historically, JAVMs were referred to as mandibular or maxillary central hemangiomas. These lesions can cause severe bleeding, either spontaneously or after minor procedures such as tooth extraction or oral surgery, and may become life-threatening. In addition, JAVMs are difficult to manage (3). Simple embolization or ligation of the feeding arteries often leads to poor outcomes and may complicate further treatment (4).

## 2 Case report

### 2.1 Case description

**Figure 1:**
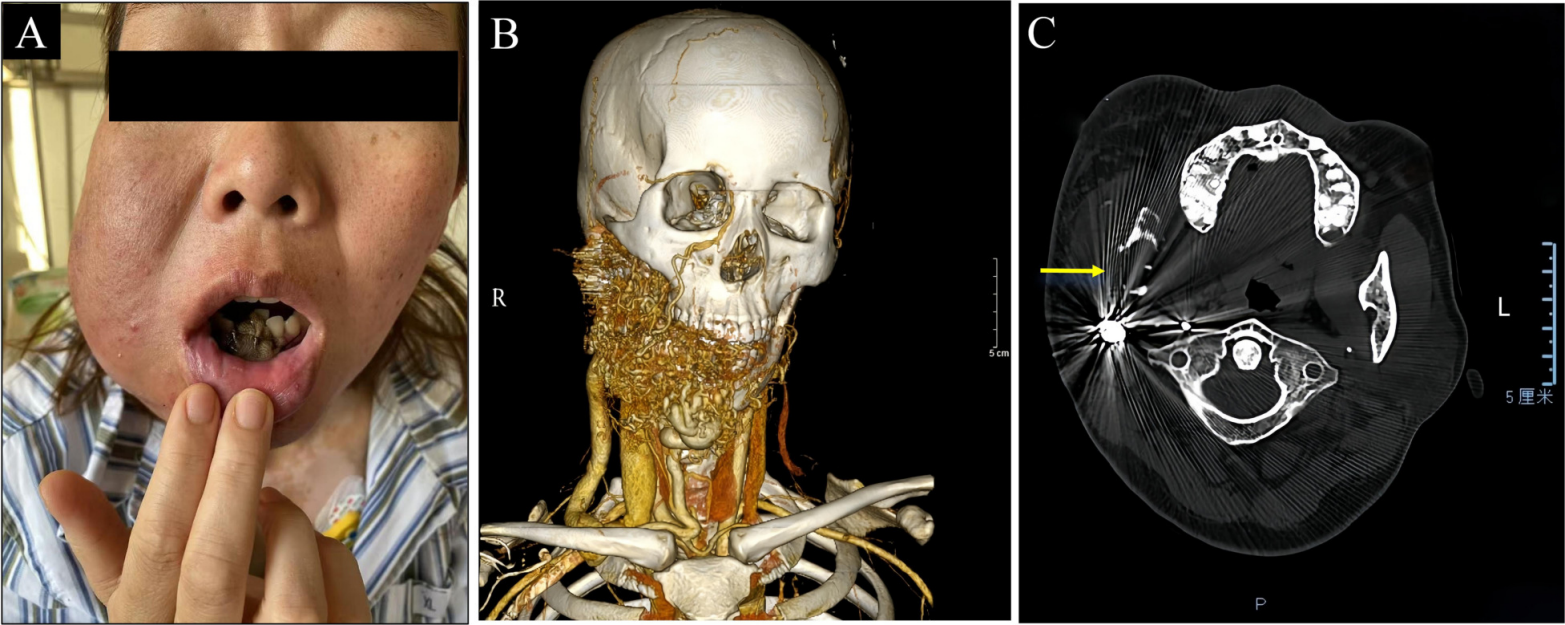
Preoperative characteristics of the patient. **(A)** Preoperative facial appearance of the patient. **(B)** Three-dimensional reconstruction of head and neck computed tomography angiography. **(C)** Head computed tomography scans.

A 33-year-old female was referred to our hospital with uncontrollable massive hemorrhage from the mandible following a tooth extraction at an outside facility. Four years prior, the patient had noticed a lesion in the right mandible, measuring approximately 10 cm at its largest diameter. The mass was soft, poorly demarcated, mildly erythematous, and tender on palpation. She had received sclerotherapy and vascular embolization at several institutions, though with limited success. In October 2023, due to recurrent gingival bleeding, she underwent extraction of anterior teeth and debridement of oral and maxillofacial soft tissues at an external hospital. However, persistent gingival hemorrhage postoperatively necessitated her transfer to our department. On clinical evaluation, the lesion was consistent with Schöbinger stage III, characterized by enlargement, pulsation, pain, ulceration, and bleeding (Figure 1A; Table 1) (5). Before transfer to our hospital, the patient's oral bleeding had already stabilized, and no further bleeding occurred during transportation. On admission, she appeared anemic, and initial laboratory tests confirmed reduced RBC, HGB, and HCT levels (Table 2). Computed tomography angiography (CTA) and other relevant investigations confirmed the diagnosis of JAVMs (Figures 1B,C).

**Table 1:** Schöbinger Stage of arteriovenous malformations (5).

Stage	Clinical findings
I (Quiescence)	Skin warmth, discoloration
II (Expansion)	Enlargement, pulsation, bruit
III (Destruction)	Pain, ulceration, bleeding
IV (Decompensation)	Cardiac failure due to volume overload

**Table 2:** Laboratory examination results at baseline and during three follow-up evaluations.

Test item	Preoperative baseline	Postoperative 2 days	Postoperative 6 months	Postoperative 1 year
Red blood cell count (RBC, ×10^12^/L)	2.92	3.21	4.62	4.83
Hemoglobin (HGB, g/L)	84	97	123	136
Hematocrit (HCT, %)	24.80	27.60	37.60	41.20

### 2.2 Treatment

**Figure 2:**
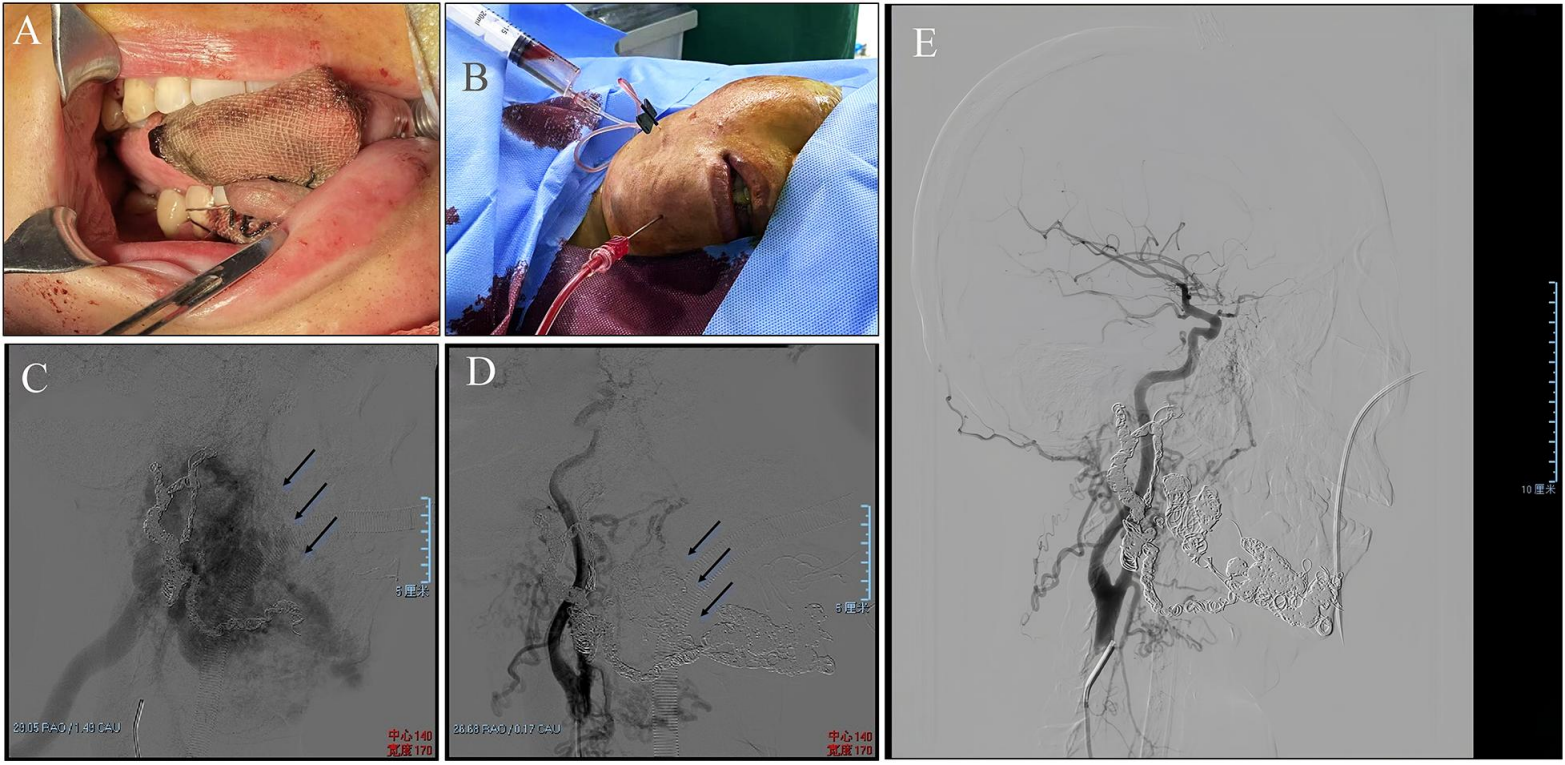
Intraoperative treatment. **(A)** Intraoral placement of a bite block and gauze for bleeding prevention. **(B)** Multiple puncture sites for treatment. **(C)** Baseline angiography during the interventional procedure. **(D)** Coil embolization and sclerosing agent injection. **(E)** Final angiography obtained at the end of the procedure.

Following preoperative assessment, the patient underwent endovascular intervention. Under general anesthesia with endotracheal intubation, we established vascular access through the right femoral artery and vein. After administering 3,000 U of heparin, angiography was performed via selective catheterization of the right carotid artery, revealing a diffuse arteriovenous fistula involving the mandible. Subsequently, a multipurpose (MP) catheter (Cordis, USA) was introduced via the femoral vein to selectively access the right internal jugular vein, confirming the presence of a diffuse arteriovenous fistula both within and over the surface of the mandible (Figure 2C).

We first punctured the most prominent area of the right mandible using an 18G micropuncture needle, followed by puncture with a 16G needle and an 18G cannula approximately 2 cm left of the mandibular midline, both of which yielded brisk blood return (Figures 2A,B). Infusion extension tubing was connected to a microcatheter system, and the microcatheter was guided into the abnormal, dilated venous cavity using a microguidewire. Embolization was then performed using 16 INTERLOCK coils (9 × 14 × 300 mm and 7 × 12 × 300 mm; Boston Scientific, USA) and 104 COOK Nester coils (Cook, Bloomington, USA), achieving dense occlusion of the venous component. Post-embolization angiography demonstrated reduced flow within the lesion. After coil embolization, endovenous sclerotherapy was performed using 16 mL of absolute ethanol and 20 mL of 3% polidocanol foam (Figure 2D). Absolute ethanol was injected directly into the nidus, with each injection being terminated when the nidus no longer appeared on angiography. The injection rate and volume were adjusted based on the AVM's flow characteristics, which were continuously monitored via angiography. To ensure safety, the total volume of ethanol injected did not exceed 1.0 mL per kilogram of the patient's body weight. Final angiography at the end of the procedure confirmed marked reduction of flow within the lesion (Figure 2E). The procedure was completed uneventfully, with hemostasis achieved through 10 min of manual compression and a pressure dressing. The patient regained full consciousness without neurological deficits and was extubated before transfer to the ward.

### 2.3 Follow-up

**Figure 3:**
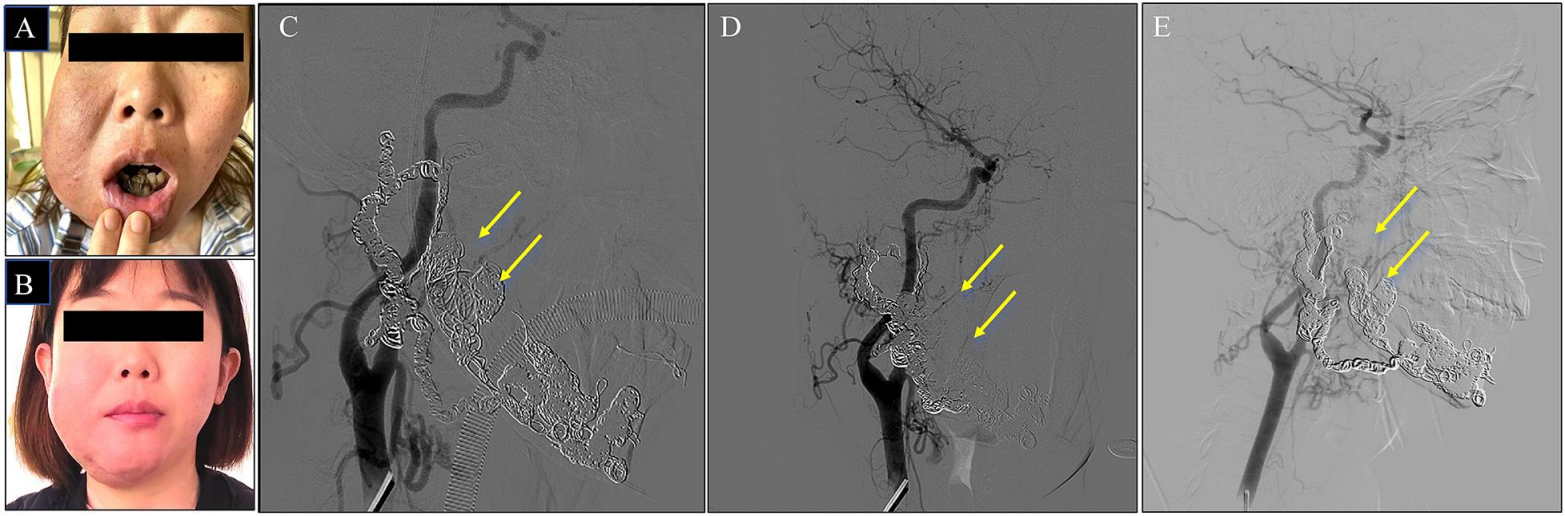
Follow-up of the patient. **(A,B)** Comparison of the patient's facial appearance before and after treatment. **(C)** Follow-up angiography at 4 days postoperatively. **(D)** Follow-up angiography at 6 months postoperatively. **(E)** Follow-up angiography at 1 year postoperatively.

The patient demonstrated favorable clinical recovery, with complete cessation of gingival bleeding. Follow-up angiography was performed on postoperative day 4, at 6 months, and again at 1 year, and laboratory test results are presented in Table 2. Imaging revealed no significant contrast enhancement within the mandible and complete resolution of the abnormal vascular cluster (Figure 3).

## 3 Discussion

AVM is a vascular disorder of relatively low incidence (6). The 5-year prevalence of simple peripheral AVM was about (2.15–6.60)/1,000,000 population (7). AVMs are frequently misdiagnosed as hemangiomas due to overlapping clinical presentations. The head and neck regions are among the most commonly involved anatomical sites, accounting for nearly 50% of all cases, followed by the extremities, trunk, and internal organs (8–10).

JAVMs represent an infrequent but clinically significant subset of congenital high-flow vascular anomalies. These lesions are characterized by abnormal arteriovenous shunting within the medullary bone and are predisposed to severe hemorrhagic events, particularly in the maxillofacial region (11). The rarity of JAVMs is underscored by the limited number of reported clinical cases. Given the intricate anatomy and rich vascularization of the mandible, these malformations are highly susceptible to iatrogenic provocation, such as tooth extractions or surgical manipulation, potentially leading to catastrophic bleeding episodes with life-threatening consequences (12).

Accurate diagnosis of JAVMs necessitates the integration of various auxiliary imaging modalities (13). However, due to their often subtle and atypical radiologic features, these lesions are prone to misdiagnosis or delayed detection (14, 15). While Doppler ultrasonography is a non-invasive and readily accessible technique, its diagnostic utility is often compromised by acoustic interference from the adjacent mandibular bone. Magnetic resonance imaging (MRI) and CTA are currently regarded as more effective diagnostic tools. MRI leverages the flow void phenomenon to delineate vascular anatomy, allowing clear distinction between feeding arteries and draining veins via time-resolved gradient echo sequences. Moreover, contrast-enhanced MRI typically shows limited enhancement in adjacent soft tissues, and its lack of ionizing radiation renders it especially suitable for pediatric and radiation-sensitive patients (16).

CTA offers detailed visualization of both the vascular components and osseous structures, contrast-enhanced CT is often more helpful in clinical practice, as it better demonstrates the relationship among the AVM lesion, the mandible, and surrounding soft tissues, thereby improving diagnostic confidence. Nevertheless, the radiation exposure and contrast-associated risks inherent in CT-based modalities warrant careful preprocedural evaluation and informed patient consent (16).

Digital subtraction angiography (DSA) is widely regarded as the diagnostic gold standard for AVMs (17). Compared to other imaging modalities, DSA allows for high-resolution, real-time assessment of the nidus, feeding arteries, draining veins, and their spatial relationships with adjacent structures. Importantly, DSA facilitates precise evaluation of lesion hemodynamics and identification of arteriovenous nidus, providing essential information for interventional planning and surgical decision-making (18). In addition, panoramic radiography is a practical supplementary tool for assessing dentition, particularly to determine whether the lesion involves tooth roots. Because JAVMs may cause bone and root of tooth resorption, identifying severely loosened teeth helps guide intraoperative extraction to reduce postoperative oral bleeding risk (19).

With the advent of minimally invasive techniques, the therapeutic landscape for mandibular AVMs has markedly evolved (20). Traditional surgical approaches, such as lesion excision and arterial ligation, are associated with significant intraoperative bleeding, high recurrence rates, and considerable postoperative morbidity (21, 22). Contemporary treatment paradigms emphasize effective hemostasis and recurrence prevention. Clinical studies report symptom control rates ranging from 82.4% to 100% with interventional embolization in hemorrhagic JAVMs (23). The crux of successful embolotherapy lies in complete occlusion of the nidus, which is pivotal for achieving long-term clinical remission (24, 25).

Recent years have seen important refinements in the use of ethanol-based embolotherapy for complex head and neck AVMs. A 10-year experience demonstrated that carefully controlled, staged ethanol injection enables deep nidus penetration and achieves durable occlusion with acceptable complication rates in high-flow vascular malformations (26). Similarly, maxillary AVMs treated using combined coil deployment and absolute ethanol achieved stable long-term outcomes, highlighting the benefits of flow-control techniques in preventing non-target embolization and minimizing recurrence (27). More recently, coil-assisted ethanol embolotherapy has proven effective in managing recurrent or refractory AVMs, including lesions demonstrating recrudescence after Onyx treatment, further underscoring the importance of individualized endovascular strategies (28).

In addition, a number of studies emphasize the value of multidisciplinary and hybrid treatment approaches for craniofacial AVMs. Surgical resection combined with sclerotherapy has been effective in anatomically complex malformations requiring structural correction (29). Ethanol embolotherapy has also demonstrated efficacy in rapidly controlling acute, life-threatening oral hemorrhage caused by mandibular AVMs and in stabilizing patients with high-output cardiac failure secondary to extensive craniofacial AVMs (30). These contemporary findings collectively highlight the shift toward tailored, multimodal treatment strategies for mandibular AVMs, aiming to reduce morbidity while achieving durable lesion control.

## 4 Conclusion

Mandibular AVMs are high-risk, challenging lesions that require accurate diagnosis and multidisciplinary, individualized treatment. Combined endovascular embolization and sclerotherapy can achieve durable lesion control, but ongoing refinement of imaging and interventional protocols and long-term surveillance remain necessary.

## Data Availability

The original contributions presented in the study are included in the article/Supplementary Material, further inquiries can be directed to the corresponding author/s.
